# Takotsubo's Cardiomyopathy in a Patient with Kartagener's Syndrome

**DOI:** 10.1155/2014/690151

**Published:** 2014-10-15

**Authors:** Luis W. Dominguez, Robert P. Doggette, Fernando Gonzalez-Ibarra, Imam H. Shaik, Amer K. Syed

**Affiliations:** ^1^Department of Internal Medicine, Jersey City Medical Center, 355 Grand Street, Jersey City, NJ 07302, USA; ^2^St. George's University School of Medicine, St. George's, Grenada; ^3^Laureate National Institute of Medicine, Internal Medicine, Jersey City Medical Center, 355 Grand Street, Jersey City, NJ 07302, USA

## Abstract

A 46-year-old African-American male with past medical history significant for Kartagener's syndrome, essential hypertension, and HIV presented with acute chest pain. ECG and troponins indicated an acute myocardial infarction. Ventriculography confirmed dyskinesia of the left ventricle, with an EF of 25%. However the coronary catheterization showed nonobstructed coronaries. Ventricular contraction and EF were restored in 4 weeks. To our knowledge, this is the first incidence of Takotsubo's reported in a young patient with Kartagener's syndrome. Chronic lung disease may contribute to the development of Takotsubo's cardiomyopathy, which is a documented yet not fully understood phenomenon.

## 1. Introduction

Takotsubo's cardiomyopathy is a transient (reversible) systolic dysfunction of the left ventricle, presenting with acute chest symptoms, electrocardiographic (ECG) changes, and elevated cardiac enzymes mimicking acute coronary syndrome (ACS) [1–8]. Left ventriculogram often reveals apical dyskinesia, the so-called apical ballooning, without any significant stenosis on coronary angiography [1, 2]. However, this is transient as systolic function normalizes within a few weeks without intervention [1, 4–6]. Takotsubo's has also been described as apical ballooning syndrome, broken heart syndrome, and stress-induced cardiomyopathy.

Sato et al. were the first to document the term Takotsubo's, describing the appearance of the left ventricle during systole [9]. Studies since then have estimated that Takotsubo's is prevalent in 1% to 2% of patients presenting with suspected ACS [10, 11]. Epidemiologically, the disease occurs mostly in postmenopausal women [8]. Prasad et al. found that 90% of reviewed cases were female and the average age of onset ranged between 58 and 75 years. This predilection for the middle-aged is notable as less than 3% of reported cases occurred in patients under 50 years of age [12]. Takotsubo's shows a heterogeneous distribution among different ethnicities. According to a literature review, 57.2% of patients were Asian and 40% were Caucasian, while only 2.8% belonged to other races [13]. Classically, Takotsubo's was believed to be associated with both physical and especially emotional stressors [1, 2, 4–7]. However, specific physical stressors such as sepsis [14] and pheochromocytoma [15] have also been identified as risk factors.

Our patient in the present case is not one of the classical cases reported in current literature and is epidemiologically rare. Whereas most cases of Takotsubo's were reported in middle-aged Asian and Caucasian females, this patient is a young African-American male. Upon admission he had neither emotional stressors nor symptomatic pheochromocytoma or sepsis. However, he had a significant history of chronic lung disease secondary to Kartagener's syndrome. The presence of chronic lung disease may trigger the development of Takotsubo's cardiomyopathy, so that such cases may further contribute to understanding the underlying pathophysiology of this entity. In addition, the lack of emotional or previously documented physiological stressors along with the atypical patient demographics for age, gender, and ethnicity makes this case remarkable.

## 2. Case Report

A 46-year-old African-American male with a past medical history significant for essential hypertension, HIV, Kartagener's syndrome with situs inversus, and pulmonary hypertension presented to the emergency department with acute chest pain. The pain was sharp, right sided, radiating to the right neck, and associated with dyspnea, nausea, and nonbloody emesis. Fever, palpitations cough, or recent trauma was not reported by the patient. He was compliant with HIV medications, was not a cigarette smoker, and reported no drug allergies or drug abuse. The patient also reported three previous partial lobectomies secondary to recurrent pneumonias over the past 25 years.

**Figure 1:**
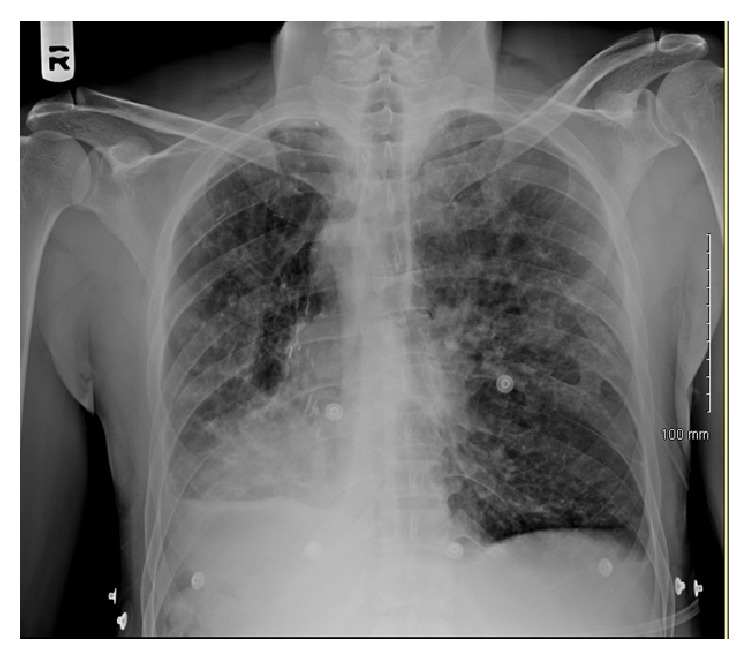
Chest radiograph showing dextrocardia and chronic interstitial markings from Kartagener's syndrome.

**Figure 2:**
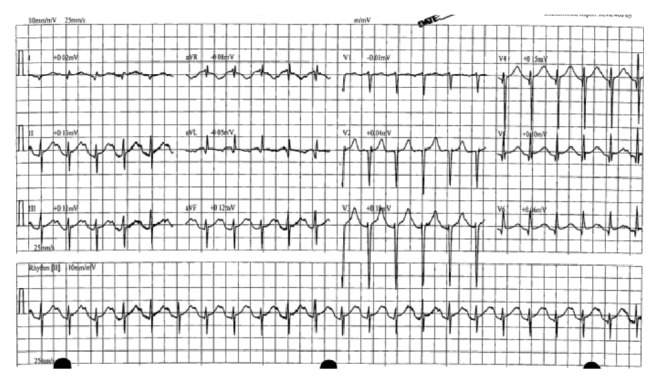
Presenting ECG showing sinus tachycardia at 136 beats/min, ST-elevations in leads V3-V6, II, III, and aVF.

Physical examination was significant for mild bilateral expiratory wheezes and dry oral mucosa, right sided apical impulse without any cardiac murmurs, jugular venous distension, or pedal edema. The patient was afebrile and blood pressure was 124/90 mmHg, heart rate at 136 beats/minute, respiratory rate of 22 breaths/min, and saturating 95% on room air. ECG showed sinus tachycardia at 136 beats/min, ST-elevations in leads V3-V6, II, III, and aVF suggestive of acute inferoseptal wall MI, as well as poor R-wave progression, associated with dextrocardia (Figure 2). Serum troponins were mildly elevated with a peak of 0.72 ng/mL. Laboratory examination revealed a mild leukocytosis (16,500 cells/mcL) with normal clinical chemistry and blood coagulation. Urine toxicology was negative for drugs. Chest X-ray demonstrated dextrocardia without signs of acute infection (Figure 1). CT angiogram showed moderate-severe bronchiectasis, dextrocardia, scarring of the right lung base, and mediastinal lymphadenopathy.

**Figure 3:**
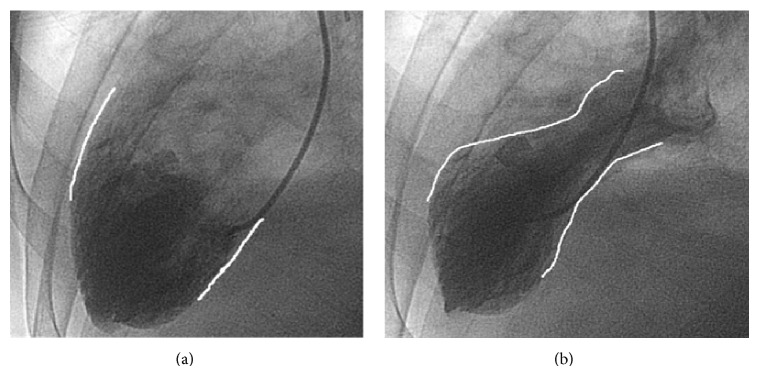
Ventriculograms showing the heart at end diastolic volume (a) and at end systolic volume (b), revealing apical ballooning seen in Takotsubo's. Outlining used for enhanced visualization of the ventricle wall for comparison.

The patient was treated with aspirin, clopidogrel, statins, beta-blockers, ACE inhibitors, and heparin for acute coronary syndrome. Nitroglycerine and morphine were also administered. Echocardiography showed dyskinesia of the left ventricular apex. Subsequent coronary angiography revealed nonobstructive coronaries, moderate dyskinesia of the apex, and mild dyskinesia of the anterolateral wall with the ejection fraction estimated at 25% on left ventriculogram (Figures 3(a) and 3(b)). The diagnosis of Takotsubo's cardiomyopathy was made based on Mayo Clinic criteria [12]. The patient was discharged 5 days later on aspirin, ACE inhibitors, beta-blockers, and statins along with a LifeVest and follow-ups scheduled at 2 and 4 weeks.

An ambulatory follow-up of the patient was scheduled after 4 weeks in order to reevaluate the need for further use of the cardioverter defibrillator. His ECG revealed normal sinus rhythm with T-wave inversions in V4-6. Echocardiogram showed normokinetic motion at the apex, and the EF was calculated at 54%, indicating complete recovery.

## 3. Discussion

Takotsubo's cardiomyopathy is defined as a transient systolic dysfunction of the left ventricle that presents as an acute MI but without significant coronary obstruction. While there are many subtypes, the classical presentation involves stunning of the apical segment, resulting in systolic dysfunction. In such cases, ECG changes are often (but not always) disproportionately significant when compared to only minimally elevated cardiac biomarkers. However, unlike an MI, Takotsubo's occurs in the absence of obstructive coronary disease on angiography [1–8, 16]. The systolic function and EF are often fully recovered within 1–4 weeks [1, 4–6]. Takotsubo's cardiomyopathy is more common in females than in males and most common in postmenopausal women [8]. The most prevalent form of Takotsubo's includes dyskinesia of the middle and apical ventricular segments [17]. Additional subtypes include dyskinesia restricted to the mid ventricle [17] or the base of the left ventricle [18]. Recent studies have also found variants that include transient right ventricular [19] and bilateral ventricular dyskinesia [20–22].

Most cases of Takotsubo's present with (1) significant ECG changes, (2) only mild troponin rise given the severity of the ECG, (3) dyskinesia of the left ventricle upon ventriculogram, and (4) the absence of obstructive coronary artery disease. The most common ECG findings are ST elevations in leads V1-V4. In addition, T-wave inversions, QT prolongation, and abnormal Q waves can also be found. Some cases have reported transient ventricular dyskinesia without any initial ECG changes [1, 5]. Cardiac biomarkers are often elevated; however it is remarkable how mild the elevation is, given the significant extent of systolic dysfunction [11]. Echocardiography and/or left ventriculogram usually reveals apical ballooning and dyskinesia of up to 66% of the LV [1, 2, 4–6, 11]. Accordingly, left ventricular ejection fraction drops precipitously from normal to as low as 20% [1, 4, 5, 11].

Two recent studies have estimated the prevalence of Takotsubo's cardiomyopathy among patients presenting with ACS to be 1.7–2.2% [11] and 1.2% [10], respectively. As this is not an insignificant number, more must be done to understand the etiology of this disease. Heightened physical and emotional stress as well as sepsis have been documented as risk factors for Takotsubo's cardiomyopathy [1, 2, 4–7]. Other studies have found both therapeutic [23] and elevated [24] levels of catecholamines as well as pheochromocytoma [15] to be associated with Takotsubo's cardiomyopathy.

Notably, Takotsubo's has also been associated with some serious complications, including left ventricular thrombi, and the associated risk for thromboembolic events. Despite the danger of strokes and other infarcts, relatively little has been published on this topic. The majority of literature is composed of case reports, with few systematic reviews. de Gregorio et al. conducted a systematic review of 14 studies finding 15 Takotsubo's patients with reported complications of left ventricular thrombi [25] (Table 1). Of the 15 patients with ventricular clots, 5 reported thromboembolic events, including 3 with stroke, indicating the risk of emboli to be 33%. According to their review, left ventricular thrombi occurred in 2.5% of all investigated Takotsubo's patients, with 0.8% accordingly reporting emboli. Haghi et al. conducted a similar study cataloguing 52 patients over a 33-month period in Germany [26]. Using echocardiography, 4 out of the 52 patients (8%) were found to have left ventricular thrombi. Of the 4 patients, all had elevated CRPs. Among the 4 patients with thrombi, 2 had clots proximate the papillary muscle while the other 2 had clots located in the ventricular apex. Over a 9-year period, Mitsuma et al. documented 21 cases of Takotsubo's. Three patients (14%) reported thromboembolic events; however only 1 patient had a visualized left ventricular thrombus [27].

Clearly, the occurrence of left ventricular thrombi requires further investigation, as the studies cited above report incidence rates ranging from 2.5% to potentially 14% of patients with Takotsubo's cardiomyopathy. Regardless, left ventricular thrombi are a documented and worrisome complication of Takotsubo's, especially due to the risk of embolic events. Fortunately, thromboemboli seem to occur less frequently than their antecedent ventricular clots. In our patient, neither ventricular thrombi nor embolic events were found.

To our knowledge, this is a rare case of Takotsubo's cardiomyopathy documented in a young male patient with Kartagener's syndrome. Furthermore, as most patients with Takotsubo's are postmenopausal, Asian, and Caucasian females, the fact that this is a relatively youthful, African-American male is remarkable. Given the current understanding of Takotsubo's as a disease resulting from transient catecholamine stress, this case offers further insight into the potential mechanism and pathophysiology of this poorly understood cardiomyopathy.

## 4. Proposed Mechanism

Kartagener's predisposes patients to chronic bronchitis and bronchiectasis. As Kartagener's progresses, scarring of the alveoli results in restrictive lung disease. Lung volume is decreased along with compliance. The resulting alveolar fibrosis inhibits gas exchange, leading to chronic hypoxia and thus pulmonary hypertension. In a similar way that sepsis or pheochromocytoma stresses the heart, the increased afterload on the RV and concurrent hypoxia with decreased preload to the LV could predispose a patient to Takotsubo's cardiomyopathy. If this is the case, hypoxia, as well as pulmonary hypertension, must be examined as possible etiologies for Takotsubo's cardiomyopathy.

## Figures and Tables

**Table 1:** Showing percentage of Takotsubo's patients with LV thrombi and the number of embolic events resulting from each thrombus.

Study	Number of LV thrombi in patients with Takotsubo's	Number of embolic events in patients with LV thrombi
de Gregorio et al. (2008) [25]	15/600 (2.5%)^a^	3/5 (60%)
Mitsuma et al. (2010) [27]	3/21 (14%)	2/3 (66%)
Haghi et al. (2008) [26]	4/52 (8%)	0/4 (0%)^b^

^a^Percentage reported in article. Total Takotsubo's cases (600) were derived from that percentage.

^b^Documented use of anticoagulants prevented embolic phenomena.
